# First principles design and band engineering of type-II As_2_C_3_/Sc_2_CF_2_ van der Waals heterostructure

**DOI:** 10.1039/d5ra07652h

**Published:** 2026-01-05

**Authors:** Nguyen Xuan Sang, Nguyen Q. Cuong, Le Phuong Long

**Affiliations:** a Atomic Molecular and Optical Physics Research Group, Institute for Advanced Study in Technology, Ton Duc Thang University Ho Chi Minh City Vietnam nguyenxuansang@tdtu.edu.vn; b Faculty of Electrical and Electronics Engineering, Ton Duc Thang University Ho Chi Minh City Vietnam; c Institute of Research and Development, Duy Tan University Da Nang 550000 Vietnam nguyenquangcuong3@duytan.edu.vn; d School of Engineering & Technology, Duy Tan University Da Nang 550000 Vietnam; e Center of Scientific Research and Application, Lac Hong University No. 10 Huynh Van Nghe Str, Tran Bien Ward Dong Nai Province Vietnam phuonglong@lhu.edu.vn

## Abstract

In this work, we systematically investigate the structural, electronic, mechanical, thermal, and optical properties of a two-dimensional (2D) As_2_C_3_/Sc_2_CF_2_ van der Waals (vdW) heterostructure using first-principles calculations. All stacking configurations preserve the semiconducting nature of their constituent monolayers with indirect bandgaps and form a type-II band alignment, favorable for efficient charge separation. The As_2_C_3_/Sc_2_CF_2_ heterostructure exhibits excellent mechanical robustness and thermal stability. Optical absorption spectra reveal a significant enhancement across a broad spectral range, with the absorption coefficient reaching up to 3.5 × 10^5^ cm^−1^. Furthermore, the electronic properties and contact behavior of the heterostructure can be effectively tuned by applying an external electric field. Notably, a semiconductor-to-metal transition is induced under negative electric field, while a reversible switching between type-II and type-I band alignments is achieved under the positive direction. These results underscore the potential of the As_2_C_3_/Sc_2_CF_2_ heterostructure as a versatile candidate for future nanoelectronic, optoelectronic, and field-effect device applications.

## Abstract 2

In this work, we systematically investigate the structural, electronic, mechanical, thermal, and optical properties of a two-dimensional (2D) As_2_C_3_/Sc_2_CF_2_ van der Waals (vdW) heterostructure using first-principles calculations.

## Graphical abstract

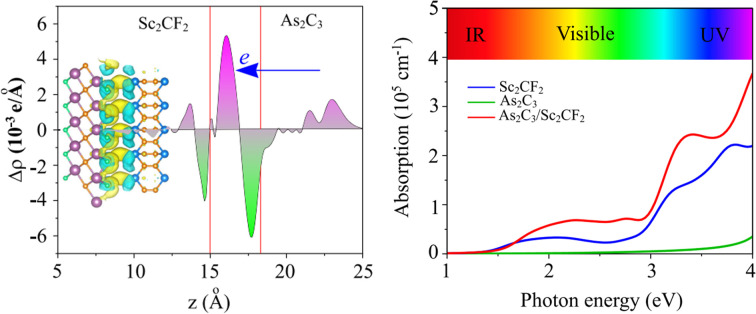

## 1 Introduction

Two-dimensional (2D) materials^1^ have emerged as a significant field of study, largely due to their remarkable electronic and optical characteristics.^2^ Since the initial isolation of graphene,^3^ research has expanded to encompass a wide array of 2D materials, including but not limited to transition metal dichalcogenides (TMDs),^4^ hexagonal boron nitride (h-BN),^5^ phosphorene,^6^ and MXenes.^7^ Among them, 2D heterostructures,^8,9^ formed by vertically stacking different monolayers, offer a promising strategy to tailor physical properties by combining the distinct characteristics of individual components. These van der Waals (vdW) heterostructures have demonstrated remarkable potential in modulating bandgap values and band alignment, both of which are crucial for applications in optoelectronics, photovoltaics, and photocatalysis.^10–12^ In particular, the band alignment type plays a pivotal role in charge carrier separation and transport, where a type-II (staggered) band alignment facilitates the spatial separation of photogenerated electron–hole pairs, enhancing device efficiency.

Recently, a variety of strategies, including external electric fields,^13,14^ mechanical strain,^15–17^ and interface engineering,^18,19^ have been employed to control the band alignment in 2D heterostructures. The ability to tune band positions and transition between different alignment types offers exciting opportunities for designing next-generation, tunable nanoelectronic and optoelectronic devices. However, accurate prediction of band alignment requires reliable theoretical investigations into the electronic structures and interfacial interactions of these heterostructures.

Among the 2D semiconductors of recent interest, arsenic carbide (As_2_C_3_) has been proposed as a dynamically stable material, with its phonon dispersion confirming mechanical and thermal stability.^20^ Density functional theory (DFT) studies have shown that monolayer As_2_C_3_ possesses a moderate bandgap of approximately 1.42/2.27 eV according to PBE/HSE06 calculations, making it highly suitable for optoelectronic and energy conversion applications.^21^ Moreover, computational predictions suggest that As_2_C_3_ exhibits high carrier mobility,^20^ indicating its promise for high-speed electronic devices. Its integration with other 2D materials has also been explored; for example, Mo *et al.*^22^ reported the formation of a type-II heterostructure between As_2_C_3_ and MoSi_2_N_4_, featuring efficient charge separation and enhanced photocatalytic performance.

Likewise, Sc_2_CF_2_,^23^ a member of the MXene family, has attracted considerable attention due to its versatile surface functionalizations^24^ and tunable electronic properties.^25,26^ Cheng *et al.*^27^ employed DFT calculations to investigate monolayer Sc_2_CF_2_ as a gas sensor material, demonstrating strong adsorption of NO_2_ molecules and significant charge transfer, underscoring the sensitivity of its electronic structure to external adsorbates. The surface termination groups (F, OH, or O) in Sc_2_CF_2_ provide a versatile platform for tailoring its electronic structure, making it an ideal candidate for band alignment engineering when forming heterostructures with other 2D materials. Several Sc_2_CF_2_-based heterostructures have been theoretically proposed, revealing diverse electronic behaviors.^28–32^ For example, the formation of a type-II band alignment in the Sc_2_CF_2_/GaN heterostructure facilitates efficient separation of photogenerated charge carriers, making it a promising candidate for photocatalytic water splitting and optoelectronic devices.^28^ Bao *et al.*^29^ predicted that Sc_2_CF_2_ demonstrates excellent photocatalytic activity for nitrogen reduction reactions (NRR) under visible light irradiation, while Li *et al.*^30^ reported its effectiveness as a visible-light-driven photocatalyst for overall water splitting, owing to its appropriate bandgap and favorable band edge alignment relative to the redox potentials of water.

While extensive theoretical work has been conducted on various 2D materials and their heterostructures, the specific electronic coupling and band alignment of the As_2_C_3_/Sc_2_CF_2_ heterostructure remain an unexplored area. This absence of research represents a crucial knowledge gap, as the precise impact of electronic interactions between these two distinct semiconducting layers on their overall band alignment and electronic structure is currently unknown. Considering the unique properties of both As_2_C_3_ and Sc_2_CF_2_, their combined system holds significant promise for exhibiting novel electronic characteristics, potentially including a beneficial type-II band alignment conducive to efficient charge separation. In this investigation, we leverage first-principles calculations to conduct a thorough examination of the structural stability, electronic properties, and band alignment of the As_2_C_3_/Sc_2_CF_2_ heterostructure. Furthermore, we delve into the effects of applying external electric fields on the band alignment type and electronic structure, thereby offering valuable theoretical guidance for the development of next-generation 2D vdW heterostructure-based electronic and optoelectronic devices.

## 2 Computational methods

Our study utilized first-principles calculations based on density functional theory (DFT), carried out with the Vienna *Ab initio* Simulation Package (VASP).^33^ To accurately describe the interactions between ions and electrons, we employed the projector augmented-wave (PAW) method.^34^ The exchange-correlation potential was handled using the generalized gradient approximation (GGA), specifically the Perdew, Burke, and Ernzerhof (PBE) parametrization.^35^ For more precise predictions of electronic properties, particularly band structures and alignments, we additionally used the Heyd–Scuseria–Ernzerhof (HSE06) hybrid functional.^36^ To ensure the reliability of our results, we set the kinetic energy cutoff for the plane-wave basis set to 400 eV and used a 9 × 9 × 1 Monkhorst–Pack *k*-point mesh for Brillouin zone integration. A 30 Å vacuum space was applied in the *z*-direction to eliminate spurious interactions from periodic images. We performed structural optimization by minimizing total energy and atomic forces until strict convergence criteria were met: an energy difference between successive steps below 10^−6^ eV and a maximum residual force per atom under 0.01 eV Å^−1^. Finally, to accurately model interlayer coupling in our 2D heterostructures, we incorporated vdW interactions using DFT-D3 method.^37^

## 3 Results and discussion

**Fig. 1:**
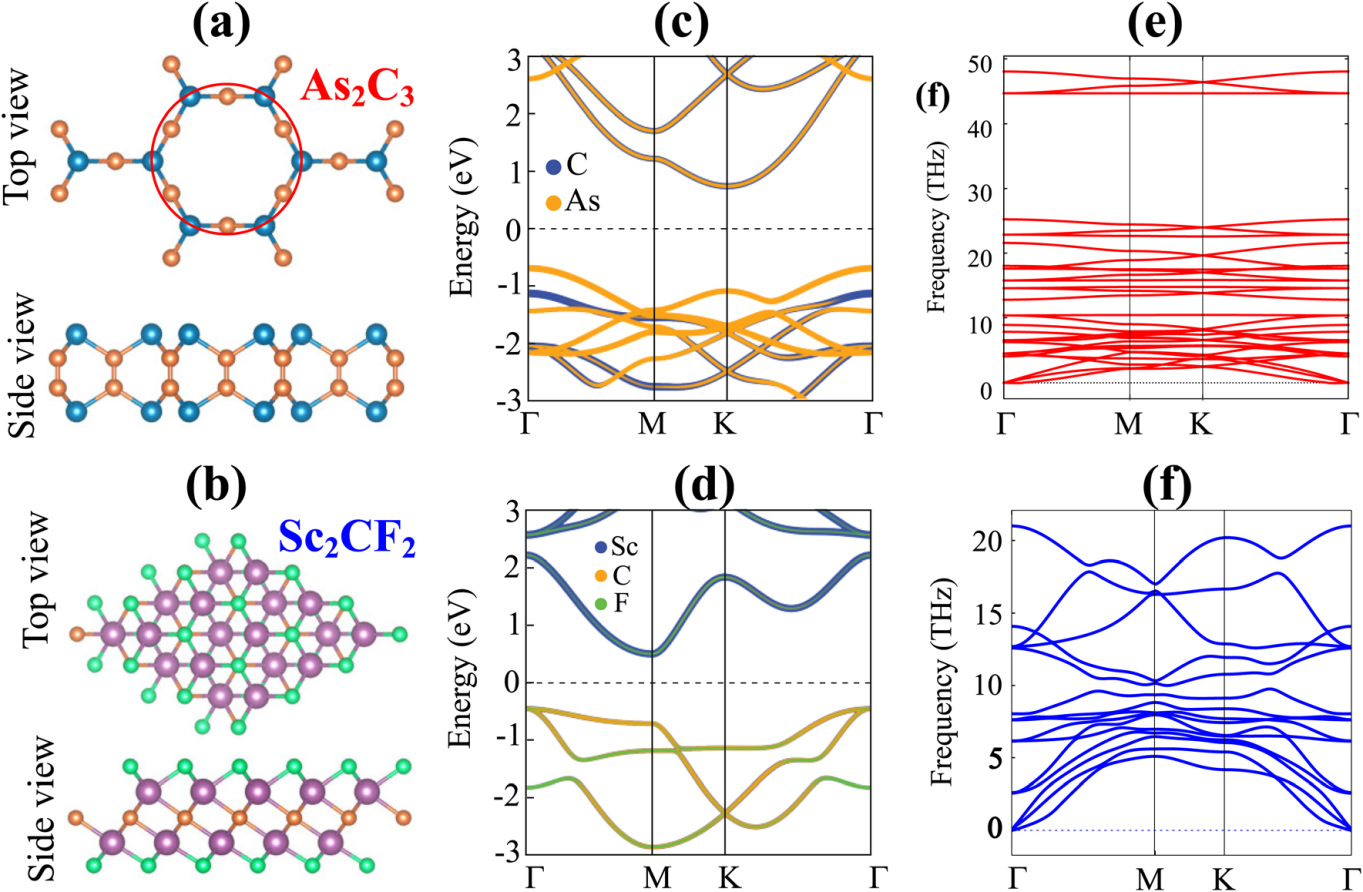
Geometric optimization of (a) As_2_C_2_, (b) Sc_2_CF_2_. Orange and cyan balls represent the C and As atoms, while green and purple balls represent the F and Sc atoms, respectively; band structures of (c) As_2_C_2_, (d) Sc_2_CF_2_ and phonon spectra of (e) As_2_C_2_, (f) Sc_2_CF_2_.

An initial investigation into the geometric and electronic properties of individual As_2_C_3_ and Sc_2_CF_2_ monolayers was performed. Monolayer As_2_C_3_ crystallizes in a hexagonal (trigonal) lattice (space group *P*3*m*1, No. 164), exhibiting an optimized in-plane lattice constant of *a* = *b* = 5.84 Å, which is in excellent agreement with earlier theoretical findings.^20,21^ The structure, shown in Fig. 1(a), features arsenic and carbon atoms forming a honeycomb framework, with each As atom covalently bonded to three adjacent C atoms. Using the PBE functional, we determined the electronic band structure of As_2_C_3_ to be that of an indirect semiconductor with a bandgap of 1.43 eV (Fig. 1(c)). The valence band maximum (VBM) originates predominantly from As-p orbitals, while the conduction band minimum (CBM) is primarily contributed by C-p orbitals. Furthermore, Fig. 1(e) illustrates the phonon dispersion of As_2_C_3_, which shows an absence of imaginary frequencies across the Brillouin zone, thus confirming the monolayer's dynamic stability.

Similarly, monolayer Sc_2_CF_2_ adopts a trigonal lattice structure with a calculated equilibrium lattice constant of *a* = *b* = 3.34 Å, consistent with previous theoretical predictions.^23,27^ The structure consists of a Sc–C–Sc sandwich layer terminated by fluorine atoms on both sides, as illustrated in Fig. 1(b). The PBE-calculated electronic band structure of Sc_2_CF_2_ indicates a semiconducting behavior with a bandgap of 0.89 eV, as shown in Fig. 1(d). The VBM is primarily derived from Sc-d orbitals, whereas the CBM is dominated by C-p and Sc-d states. This suggests strong hybridization between Sc and C atoms near the Fermi level. The absence of imaginary phonon modes further confirms the dynamical stability of the Sc_2_CF_2_ monolayer (see Fig. 1(f)). Furthermore, the HSE06 hybrid functional was employed to obtain a more accurate estimation of the band gaps of the investigated 2D materials. The calculated HSE06 band gaps for the As_2_C_3_ and Sc_2_CF_2_ monolayers were found to be 2.30 and 1.96 eV, respectively. Although the PBE and HSE06 approaches yield quantitatively different band gap values, both functionals consistently indicate that As_2_C_3_ and Sc_2_CF_2_ possess semiconducting characteristics. Therefore, the PBE functional is adopted for subsequent investigations.

**Fig. 2:**
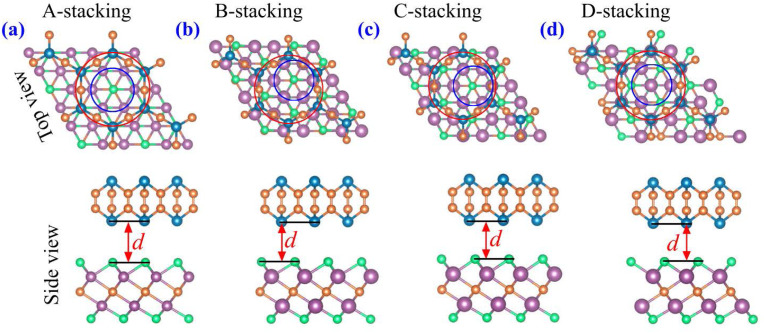
Atomic structures of the As_2_C_3_/Sc_2_CF_2_ heterostructure, presented in top and side views for (a) A-stacking, (b) B-stacking, (c) C-stacking, and (d) D-stacking configurations. Carbon and arsenic atoms are shown as orange and cyan spheres, respectively, while fluorine and scandium atoms are represented by green and purple spheres.

To explore the potential formation of a stable vdW heterostructure, we constructed four different stacking configurations of the As_2_C_3_/Sc_2_CF_2_ heterostructure by varying the relative lateral positions of the two monolayers. These configurations are labeled as stacking A, B, C and D, as depicted in Fig. 2. For each configuration, full structural optimization was performed while keeping the lattice constant fixed to the average value of the two constituent monolayers to minimize lattice mismatch and interfacial strain effects. To construct a As_2_C_3_/Sc_2_CF_2_ heterostructure with minimal lattice mismatch, a 3 × 3 supercell of Sc_2_CF_2_ was matched with a 
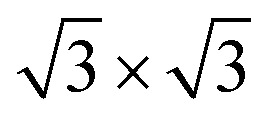
 supercell of As_2_C_3_ with the transformation matrix as follows:
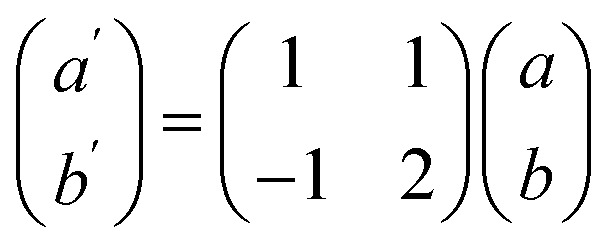
1
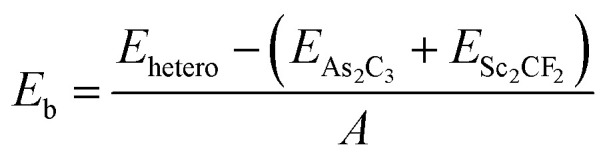
where *E*_hetero_ is the total energy of the heterostructure, *E*_As_2_C_3__ and *E*_Sc_2_CF_2__ are the total energies of the isolated monolayers within the same supercell size, and *A* is the supercell area of the heterostructure. A more negative binding energy indicates a more energetically favorable stacking configuration. The interlayer distance *d* for the stacking A, B, C and D are 3.67, 3.48, 3.50 and 3.32 Å, respectively. Meanwhile, the corresponding binding energies are evaluated to be −7.65, −9.24, −9.08 and −10.67 meV Å^−2^, respectively for stacking A, B, C and D. These values are comparable to those reported for other typical vdW heterostructures, such as graphite with an interlayer distance of 3.35 Å and binding energy of −17 meV Å^−2^,^38^ MoTe_2_/SnSe_2_ heterostructure with 3.28 Å and −17.4 meV Å^−2^,^39^ WSSe/BSe heterostructure with 3.36 Å and −18.6 meV Å^−2^.^40^ Furthermore, our calculated parameters fall within the same energetic and structural regime as those observed in previously reported As_2_C_3_-based^22,41^ and Sc_2_CF_2_-based heterostructures.^28,29,42,43^ Taken together, these comparisons demonstrate that the As_2_C_3_/Sc_2_CF_2_ heterostructure exhibits stable interlayer coupling that is fully comparable to those of related heterostructures constructed from either As_2_C_3_ or Sc_2_CF_2_ monolayers, and may even offer more favorable structural and energetic characteristics in certain configurations. These results confirm that the As_2_C_3_/Sc_2_CF_2_ system is governed by weak vdW forces. Among these, D-stacking exhibits the most negative binding energy and the shortest interlayer distance, indicating that it is the most energetically stable configuration. Therefore, this configuration was selected for subsequent electronic structure and band alignment investigations.

**Fig. 3:**
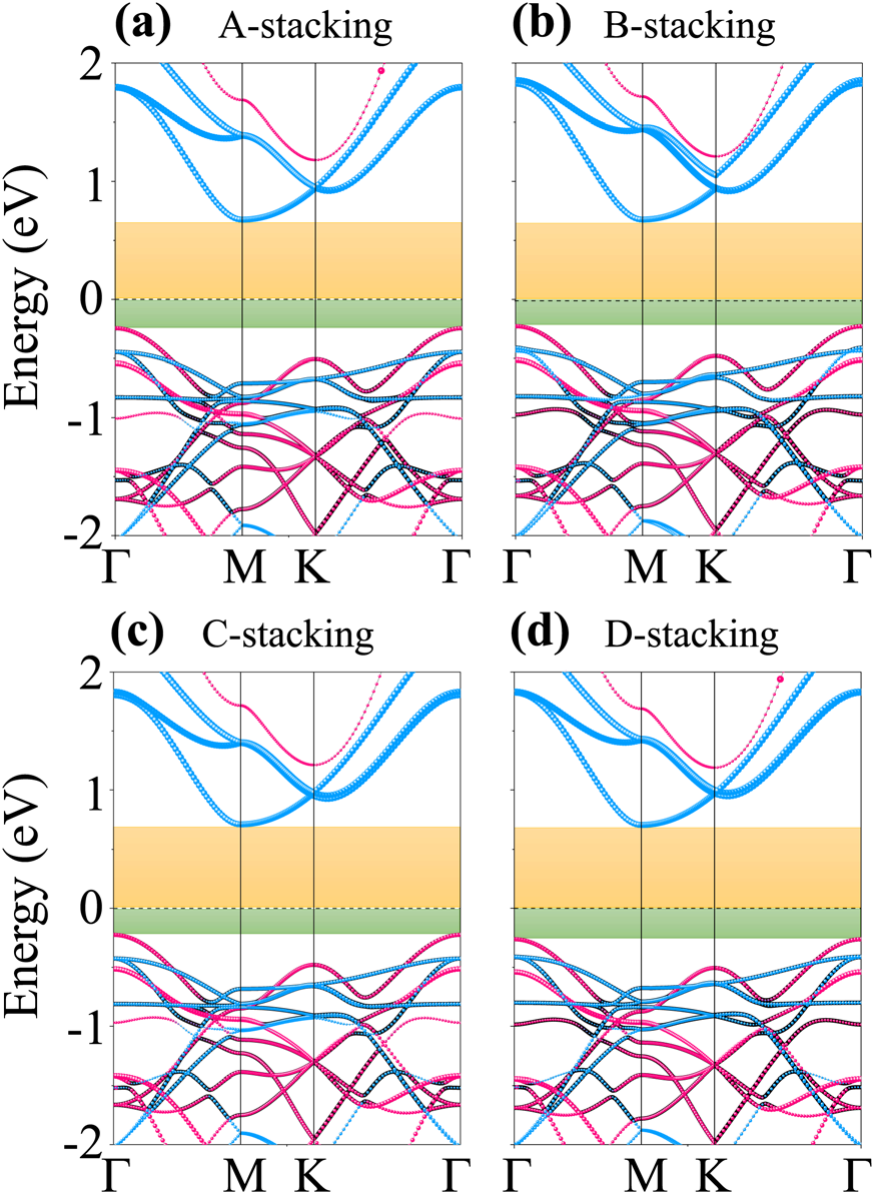
Projected band structures of the As_2_C_3_/Sc_2_CF_2_ heterostructure for different stacking configurations of (a) A-stacking, (b) B-stacking, (c) C-stacking and (d) D-stacking. Violet and cyan lines represent the projections of As_2_C_3_ and Sc_2_CF_2_ layers, respectively. Green and yellow shaded regions highlight the band-edge positions corresponding to the VBM of As_2_C_3_ layer and CBM of the Sc_2_CF_2_ layer, respectively.

This supercell combination results in a negligible lattice mismatch of approximately 0.17%. The interlayer distances *d* between the two constituent monolayers and the binding energies *E*_b_ for each stacking configuration were calculated to evaluate their relative stability. The interlayer binding energy was computed using the following formula:

To further elucidate the electronic properties of the As_2_C_3_/Sc_2_CF_2_ heterostructures, we systematically analyze the electronic band structures for all four stacking configurations, as presented in Fig. 3. As observed, the band structures of all heterostructures essentially preserve the electronic characteristics of their constituent monolayers, confirming by a weak vdW interaction. Specifically, the projected band structures can be viewed as a combination of the individual electronic states of As_2_C_3_ and Sc_2_CF_2_. Importantly, all four stacking configurations exhibit semiconducting behavior with indirect bandgaps. The calculated bandgap values slightly vary with stacking sequence, ranging from 0.92 to 0.96 eV. Furthermore, the VBM at the Γ point of heterostructure originates from the As_2_C_3_ layer, while the CBM arises from the Sc_2_CF_2_ layer for all four stacking configurations. This spatial separation of the band edges confirms the formation of a type-II band alignment in the As_2_C_3_/Sc_2_CF_2_ heterostructure, which is highly desirable for applications in photovoltaics and photocatalysis due to the efficient separation of photoexcited electron–hole pairs.

**Fig. 4:**
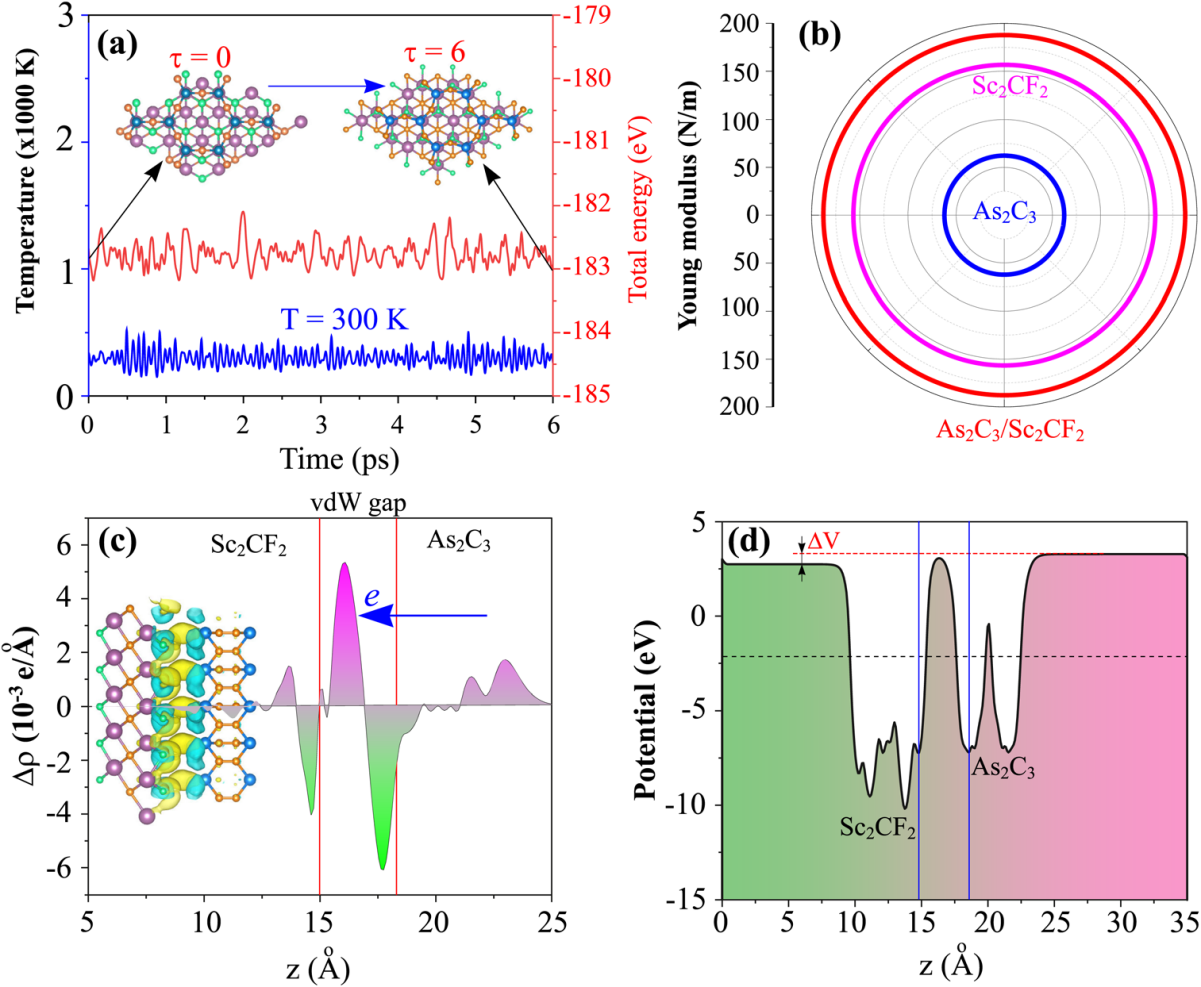
(a) AIMD simulation of the fluctuations in the temperature and total energy, (b) angular dependence of the Young modulus, (c) charge density difference and (d) electrostatic potential of the most energetically favorable stacking configuration of the As_2_C_3_/Sc_2_CF_2_ heterostructure.

To evaluate the thermal stability of the most stable D-stacking configuration, *ab initio* molecular dynamics (AIMD) simulations were performed at a temperature of 300 K for a duration of 6 ps with a time step of 1 fs, employing the NVT ensemble with a Nose–Hoover thermostat. As shown in Fig. 4(a), the temperature and total energy of the heterostructure exhibits small fluctuations around a constant average value throughout the simulation period, with no abrupt energy changes or structural reconstructions observed. Furthermore, the final atomic configuration after the AIMD simulation remains intact without any visible bond breaking, layer delamination, or significant atomic displacements. These results clearly demonstrate the excellent thermal stability of the As_2_C_3_/Sc_2_CF_2_ heterostructure under moderate thermal conditions, confirming its potential viability for practical applications in nanoelectronic and optoelectronic devices. Subsequently, to further evaluate the mechanical stability and in-plane stiffness of the most energetically favorable configuration (D-stacking) of the As_2_C_3_/Sc_2_CF_2_ heterostructure, we calculate the elastic constants by applying small in-plane strains and deriving the corresponding stress tensors. For a 2D trigonal system, the elastic constants *C*_*ij*_ should satisfy the Born stability criteria:

2 *C*_11_ > 0, *C*_66_ > 0, *C*_11_ − *C*_12_ > 0

The elastic constants for the D-stacking configuration are obtained to be *C*_11_ = 220.12 N m^−1^, *C*_12_ = 84.50 N m^−1^ and *C*_66_ = 67.81 N m^−1^. The results confirm that all mechanical stability conditions are fulfilled, verifying the mechanical robustness of the heterostructure. Notably, the heterostructure demonstrates improved in-plane stiffness relative to its isolated monolayers, suggesting enhanced resistance to in-plane strain. From a mechanical perspective, vdW heterostructures with superior elastic constants are advantageous as potential supporting or substrate materials. In such systems, the weak vdW interlayer interactions can serve as an effective mechanism to mediate and transfer strain between layers, enabling controlled deformation in monolayers with comparatively lower mechanical rigidity. To further understanding of the mechanical anisotropy of the most stable As_2_C_3_/Sc_2_CF_2_ heterostructure (D-stacking), we performed the angular dependence of the in-plane Young's modulus as follows:3
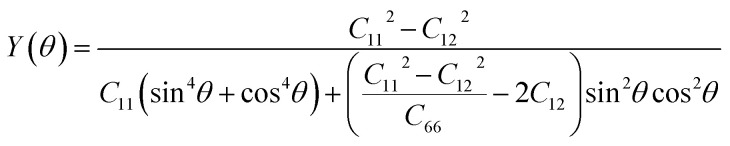


The calculated Young modulus values are illustrated in polar coordinates in Fig. 4(b), showing an isotropic in-plane mechanical response. The calculated maximum Young's modulus reaches 187.68N m^−1^, which is notably higher than that of the individual monolayers, with 62.15 N m^−1^ for As_2_C_3_ and 156.61 N m^−1^ for Sc_2_CF_2_. This enhancement indicates that the formation of the vdW heterostructure improves the in-plane mechanical stiffness while preserving isotropy.

To gain deeper insight into the interfacial charge redistribution and the nature of interlayer interactions within the As_2_C_3_/Sc_2_CF_2_ heterostructure, we computed the charge density difference (CDD) as below:

4 Δ*ρ*(**r**) = *ρ*_hetero_(**r**) − *ρ*_As_2_C_3__(**r**) − *ρ*_Sc_2_CF_2__(**r**)

where *ρ*_hetero_(**r**) represents the total charge density of the heterostructure, while *ρ*_As_2_C_3__(**r**) and *ρ*_Sc_2_CF_2__(**r**) denote the charge densities of the isolated As_2_C_3_ and Sc_2_CF_2_ monolayers, respectively. As depicted in Fig. 4(c), the CDD map reveals evident charge redistribution at the interface. Notably, regions of charge depletion (negative Δ*ρ* values) are predominantly located near the As_2_C_3_ layer, while regions of charge accumulation (positive Δ*ρ* values) are observed in proximity to the Sc_2_CF_2_ layer. This behavior indicates a net charge transfer from the As_2_C_3_ monolayer toward the Sc_2_CF_2_ monolayer upon heterostructure formation. Such interfacial charge redistribution can be attributed to the difference in work functions between the constituent monolayers, driving electrons from the relatively higher Fermi level material (As_2_C_3_) toward the lower Fermi level material (Sc_2_CF_2_) until an interfacial equilibrium is established, as illustrated in the electrostatic potential in Fig. 4(d). The charge transfer not only stabilizes the vdW heterostructure but also contributes to the formation of a built-in electric field across the interface, which is beneficial for efficient separation of photogenerated charge carriers in potential optoelectronic applications. To quantify the interfacial charge transfer, the cumulative charge transfer *Q*(*z*) was evaluated by integrating the charge density difference along the *z*-direction:5
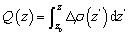
As shown in Fig. 4(c), the total amount of transferred charge is estimated to be approximately 0.003*e* per supercell. This relatively small charge transfer indicates that the As_2_C_3_/Sc_2_CF_2_ heterostructure is predominantly governed by weak vdW interactions, with minimal interlayer orbital hybridization.

**Fig. 5:**
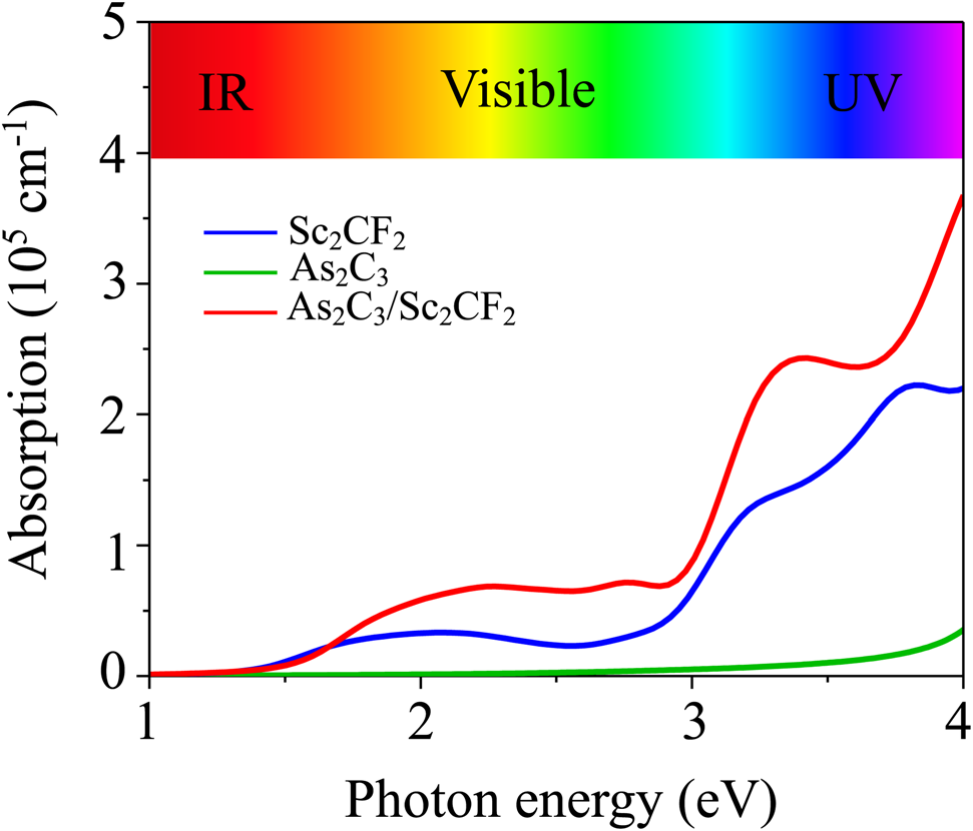
Absorption coefficient of the As_2_C_3_/Sc_2_CF_2_ heterostructure for the most energetically favorable stacking configuration.

Furthermore, optical absorption plays a crucial role in evaluating the application potential of a heterostructure, as it reflects the efficiency with which the material interacts with and harvests incident photons. The optical absorption of the heterostructure can be obtained as follows:6
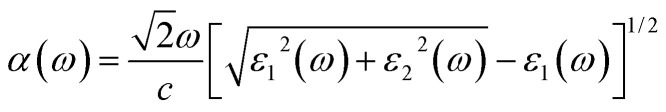
where *ε*_1_(*ω*) and *ε*_2_(*ω*) are the real and imaginary parts of the dielectric function, respectively, *ω* is the photon energy, and *c* is the speed of light. It should be noted that the Bethe–Salpeter equation (BSE) method provides a more accurate and rigorous description of optical absorption spectra, particularly for systems where excitonic effects play an important role.^44–46^ However, performing BSE calculations for the present As_2_C_3_/Sc_2_CF_2_ heterostructure involves extremely high computational cost due to the relatively large supercell and the need for dense *k*-point sampling to achieve convergence. Hence, in this work, we used the DFT-based approach to obtain the optical absorption spectra, which allows us to reliably capture the essential optical features of the heterostructure while maintaining computational efficiency. As depicted in Fig. 5, the As_2_C_3_/Sc_2_CF_2_ heterostructure exhibits a pronounced enhancement in optical absorption relative to its constituent monolayers across a broad spectral range, encompassing the infrared (IR), visible, and ultraviolet (UV) regions. Remarkably, the absorption coefficient reaches approximately 2.5 × 10^5^ cm^−1^ in the visible range and increases further to about 3.5 × 10^5^ cm^−1^ in the UV region. This substantial optical response suggests strong light–matter interactions and highlights the potential of the heterostructure for optoelectronic and photonic applications.

**Fig. 6:**
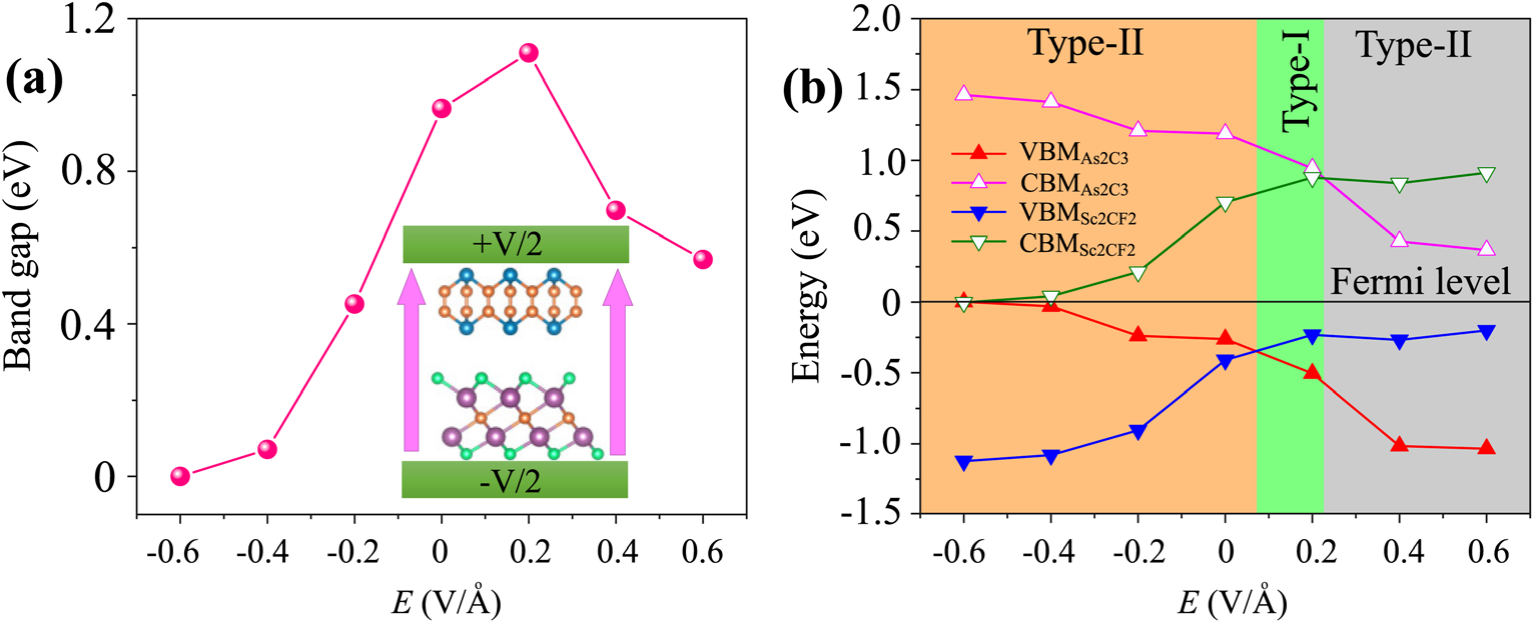
(a) The variations in the band gap and (b) band edges of the As_2_C_3_/Sc_2_CF_2_ heterostructure under applied *E*.

**Fig. 7:**
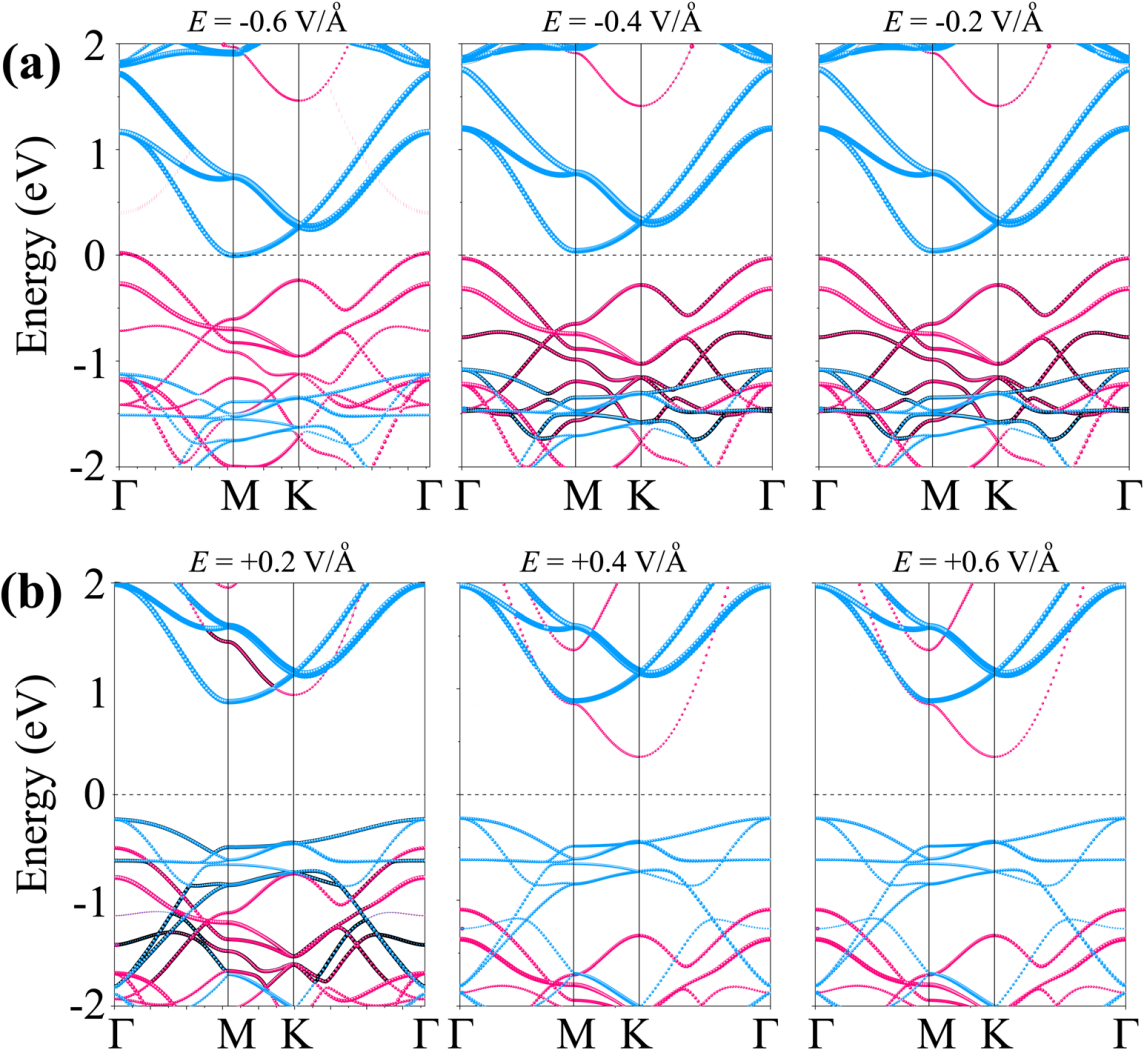
Projected band structure of the As_2_C_3_/Sc_2_CF_2_ heterostructure under (a) negative and (b) positive *E*. Purple and cyan lines present the projections of As_2_C_3_ and Sc_2_CF_2_ layers, respectively.

Furthermore, the application of an external electric field is a well-established and effective strategy for tuning the electronic properties of two-dimensional (2D) materials and their van der Waals heterostructures. Therefore, investigating the impact of an external electric field (*E*) on the As_2_C_3_/Sc_2_CF_2_ heterostructure is essential for gaining insights into its tunable electronic behavior and assessing its feasibility for future field-effect nanoelectronic and optoelectronic devices. In this study, *E* is applied perpendicular to the heterostructure, directed from the Sc_2_CF_2_ layer toward the As_2_C_3_ layer, as illustrated in the inset of Fig. 6(a). As shown in Fig. 6, the bandgap of the heterostructure demonstrates a pronounced dependence on both the magnitude and direction of the applied electric field. Specifically, under a negative electric field, the bandgap decreases monotonically with increasing field strength. In contrast, a positive electric field initially causes the bandgap to widen, reaching a maximum at approximately +0.2 V Å^−1^. However, upon further increasing the positive field strength beyond this threshold, the bandgap begins to decrease. This asymmetric and nonlinear modulation behavior can be attributed to the shifting of band edges in response to the external *E*, as depicted in Fig. 6(b) and 7. It is evident that, as the external electric field *E* is varied from −0.6 to +0.6 V Å^−1^, the energy positions of the band edges associated with the As_2_C_3_ layer progressively shift downward relative to the Fermi level. In contrast, the band edge positions of the Sc_2_CF_2_ layer exhibit an upward shift under the same field variation. Notably, the application of a negative electric field drives the heterostructure toward a semiconductor-to-metal transition due to the closure of the bandgap. In contrast, applying a positive electric field modulates the band alignment, enabling a reversible transition between type-II and type-I band alignment. Such reversible switching between type-II and type-I band alignment makes the As_2_C_3_/Sc_2_CF_2_ heterostructure highly promising for multifunctional optoelectronic applications. This electric field-induced band alignment tunability thus provides a versatile platform for next-generation, electric field-controllable 2D heterostructure-based devices.

Furthermore, the projected band structures of the As_2_C_3_/Sc_2_CF_2_ heterostructure under various external electric fields were examined to elucidate the underlying mechanism governing the observed bandgap modulation. As shown in Fig. 7(a), applying a negative *E* causes the band edges of the As_2_C_3_ layer to shift upward in energy, while the corresponding band edges of the Sc_2_CF_2_ layer shift downward. Conversely, under a positive electric field, the band edges of As_2_C_3_ move downward, whereas those of Sc_2_CF_2_ shift to higher energies, as illustrated in Fig. 7(b). Moreover, both the VBM and CBM of the As_2_C_3_/Sc_2_CF_2_ heterostructure at the negative *E* of −0.6 V Å^−1^ cross the Fermi level, indicating the occurrence of a semiconductor-to-metal transition. In contrast, under positive electric fields, the relative displacement of the band edges not only modulates the band gap magnitude but also alters the band alignment type. Specifically, a transition from type-II to type-I band alignment is observed at the positive fields (around +0.2 V Å^−1^), followed by a reversion to type-I alignment at positive *E*. Notably, at a positive field of +0.2 V Å^−1^, both the VBM at the Γ point and the CBM at the M point originate from the Sc_2_CF_2_ layer, indicating a transition from type-II to type-I band alignment. The electric-field–induced type-II to type–I transition originates from the asymmetric interlayer potential modulation, which causes opposite shifts in the band edges of the constituent monolayers. At approximately +0.2 V Å^−1^, the downward shift of the Sc_2_CF_2_ states and the upward shift of As_2_C_3_ states become sufficiently pronounced that both the VBM and CBM are localized within the Sc_2_CF_2_ layer, thereby producing a type-I band alignment. Thus, the underlying mechanism is attributed to the electric-field–driven redistribution of the electrostatic potential across the interface, which selectively alters the band edges and facilitates the field-tunable realignment of electronic states. However, upon further increasing the positive field strength, the CBM shifts to the K point and is contributed by the As_2_C_3_ layer, while the VBM remains localized in the Sc_2_CF_2_ layer. This re-separation of the band edges across different layers marks a reversion back to type-II alignment. Such electric-field-induced reversible switching between type-II and type-I band alignments, coupled with semiconductor-to-metal transitions highlights the remarkable tunability of the electronic structure and contact behavior in the As_2_C_3_/Sc_2_CF_2_ heterostructure, an essential feature for future applications in tunable optoelectronic and nanoelectronic devices.

## 4 Conclusions

In summary, comprehensive first-principles calculations have been conducted to explore the structural, electronic, mechanical and optical properties of the two-dimensional As_2_C_3_/Sc_2_CF_2_ van der Waals heterostructure. The As_2_C_3_/Sc_2_CF_2_ heterostructure was identified as the most stable structure, demonstrating excellent mechanical and thermal stability. The obtained results reveal that all stacking configurations retain the semiconducting nature of the constituent monolayers, exhibiting indirect bandgaps and forming type-II band alignment, which is advantageous for facilitating charge carrier separation in optoelectronic applications. Moreover, the As_2_C_3_/Sc_2_CF_2_ heterosrtructure exhibits a remarkable enhancement in absorption intensity with a maximum value reaching 3.5 × 10^5^ cm^−1^ in the ultraviolet region. Specially, the application of an external electric field was demonstrated as an effective approach to modulate the electronic properties of the heterostructure. The bandgap exhibited a strong dependence on both the strength and direction of the applied field, with a semiconductor-to-metal transition observed under a negative electric field. Notably, a reversible switching between type-II and type-I band alignments was achieved under moderate positive fields, offering valuable tunability for field-effect and optoelectronic device designs. These findings highlight the As_2_C_3_/Sc_2_CF_2_ heterostructure as a promising candidate for future applications in next-generation nanoelectronics, optoelectronics, and tunable photonic devices.

## Conflicts of interest

The authors declare no competing financial interest.

## Data Availability

The data that support the findings of this study are available from the corresponding author upon reasonable request.
